# 1q jumping translocation as a biomarker in myeloid malignancy: frequently mutated genes associated with bad prognosis and low survival

**DOI:** 10.1186/s40164-024-00541-3

**Published:** 2024-08-01

**Authors:** Eitan Halper-Stromberg, Victoria Stinnett, Laura Morsberger, Aparna Pallavajjala, Mark J. Levis, Amy E. DeZern, Michelle Lei, Brian Phan, Rena R. Xian, Christopher D. Gocke, Guilin Tang, Ying S. Zou

**Affiliations:** 1 Department of Pathology and Laboratory Medicine, Perelman School of Medicine at the University of Pennsylvania, Philadelphia, PA USA; 2 Departments of Pathology and Oncology, Johns Hopkins University School of Medicine, Baltimore, MD USA; 3 Department of Hematopathology, The University of Texas MD Anderson Cancer Center, 6565 MD Anderson Blvd, Houston, TX 77030 USA

**Keywords:** Gene mutation, 1q jumping translocation, Prognosis, Myeloid malignancy

## Abstract

1q jumping translocation (JT) is rare and its molecular profiles in myeloid malignancies are not well-known. This study evaluated gene mutations in 1q-JT cohorts (0.38%) from hematological malignant specimens that underwent genetic analysis at the Johns Hopkins Hospital (*n* = 11,908) and the MD Anderson Cancer Center. 1q-JT had frequent mutations in eleven genes, most of which are associated with worse prognosis. *BCOR* mutations significantly co-occurred with others. Patients tended to have mutations in DNA-repair, spliceosome, and epigenetic modification pathways, though genes utilized within each of these pathways were not randomly distributed. Multi-, albeit overlapping, pathway interruptions tended to manifest in mutations of two gene sets. One gene set consisted of *SF3B1* (spliceosome) and *TET2* (epigenetic modification), while the other consisted of *STAG2* (DNA repair), *SRSF2*, *U2AF* (spliceosome), *ASXL1*, *KMT2D* (epigenetic modification), *BCOR*, and *GATA2* (transcription factors). An “intermediate” JT-like rearrangement may represent an early sign of occurring 1q-JT. Treatments (hypomethylating agents) and unique structures of the short arms of acrocentric chromosomes may contribute to 1q-JT formation in myeloid malignancies. The median overall survival after identification of a JT was 10 months (95% confidence interval, 5–15 months). Our cohort represents the largest number of myeloid malignancies from multi-centers with before and after the 1q-JT event analyzed to date. Overall, this study identified specific molecular profiles that are associated with 1q-JT in myeloid malignancies. 1q-JT could serve as a poor prognosis biomarker in myeloid malignancies, which could be important in making well-informed clinical decisions and treatment strategies.

**Supplementary Information:**

The online version contains supplementary material available at 10.1186/s40164-024-00541-3.

## To the editor

Jumping translocation (JT) is a rare chromosomal rearrangement comprising one donor and multiple recipient chromosomes [1]. JTs involve bands 1q12-q21 as the donor segment, referred to as 1q-JT, which have been reported in ~ 50 myeloid malignancies and only a few patients had mutation data [2–5]. Given the rarity of 1q-JTs in myeloid malignancies and lack of large case series, the molecular profiles of 1q-JT cases in myeloid malignancies are not well-known.

**Fig. 1:**
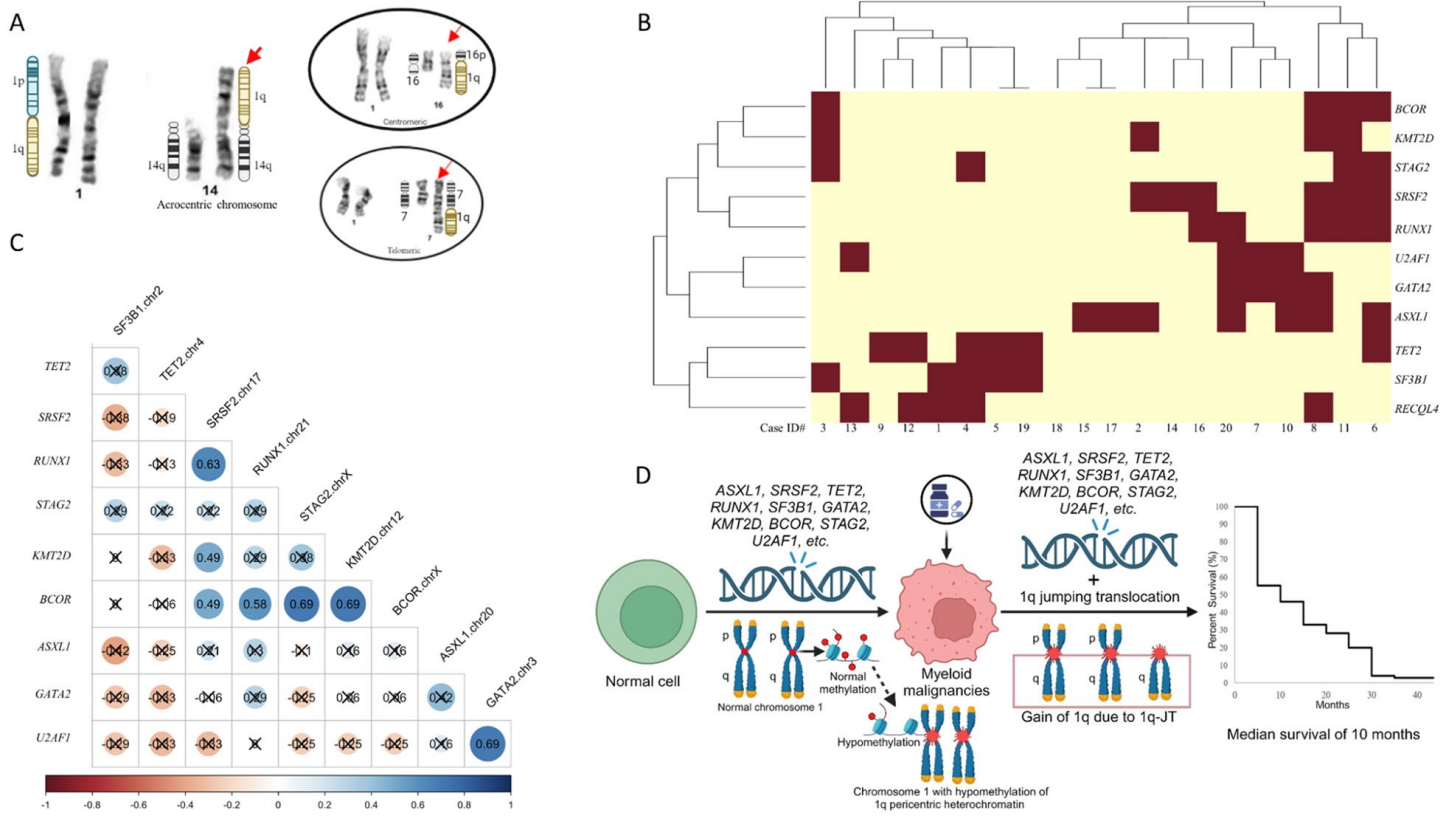
**Genetic data and survival curve of jumping translocation (JT) cases in this study. (A)** Various donor chromosome regions involved in 1q-JT formation. Short arms of acrocentric chromosomes (including 13p, 14p, 15p, 21p, and 22p) are frequently involved in 1q-JT formation, while other genomic regions (such as centromeric and telomeric regions) are infrequently involved in 1q-JT formation. Left is a partial karyogram of case #2 with 1q JT to a short arm of acrocentric chromosome 14 (red arrow). Right top insert is a partial karyogram of case #6 with 1q JT to a centromeric region of 16q and right bottom insert is a partial karyogram of case #3 with 1q JT to the telomere region of chromosome 7q (red arrows). **(B)** Heat map of the common mutated genes in the 1q-JT (Johns Hopkins) cohort. The most frequently mutated genes, in descending order, were *ASXL1*, *SRSF2*,* TET2*, *RUNX1*,* RECQL4*,* SF3B1*, *GATA2*,* KMT2D*,* BCOR*,* STAG2*, and *U2AF1*. While *RECQL4* were among the most frequently mutated gene in this study, all variants in *RECQL4* were of unknown clinical significance and favored germline. Cluster analysis of these genes generated two groups, one tending towards mutations in *SF3B1* and *TET2* (first 8 columns on the left side of Fig. 1**B**) and the other tending towards mutations in *BCOR*,* KMT2D*, *STAG2*, *SRSF2*, *RUNX1*, *U2AF1*, *GATA2*, and *ASXL1* (Fig. 1**B**), though mutations were not mutually exclusive across groups. Specimens lacked mutation in *NPM1*, a frequently mutated gene in AML, associated with better prognosis. *FLT3*-ITD, a mutation associated with worse prognosis, was detected in one of the 20 patients. **(C)** Among the most frequently mutated genes, the most frequently significantly co-mutated gene (q-value < 0.05 based on Benjamini-Hochberg false discovery rate control of P-values from Pearson correlation tests) was *BCOR*. *BCOR* was significantly co-mutated with *KMT2D*, q-value = 0.01, and *STAG2*, q-value = 0.01 (numbers in circles indicate Pearson correlation coefficients, values obscured with an 'X' have P-values >=0.05). Two other pairs of genes were significantly co-mutated, *U2AF1* and *GATA2* q-value = 0.01, and *RUNX1* and *SRSF2*, q-value = 0.04. **(D)** Diagram of potential mechanisms associated with development of 1q-JT in myeloid malignancies. Pre 1q JT patients with myeloid malignancies had these frequent mutations and may treat with hypomethylating agents (HMAs) such as azacitidine and decitabine before JT. HMAs may lead to epigenetic alteration such as hypomethylation of chromosome 1q12 pericentric heterochromatin, which may contribute to development of a double-strand break. Short arms of acrocentric chromosomes are frequently involved in 1q-JT formation because their distinctive genomic structure with centromere sequences and no coding genes makes them well-known for chromosomal rearrangements/ recombination. The presence of these pre JT mutations and gain of 1q due to JT led to a poor survival in these 1q-JT patients with myeloid malignancies. The median overall survival after JT occurrence was 10 months (95% confidence interval, 5–15 months) in this study

We reviewed 11,908 hematological malignant specimens that underwent karyotyping performed from 2016 to 2023. 46 (~ 0.38%) specimens from 21 patients had myeloid malignancies and 1q-JT (Table 1, S1, Fig. 1A). Across 56 specimens (19 pre and 37 post JT) from 20 patients with concurrent deep Next-Generation-Sequencing (NGS, supplementary methods [6]), we observed 45 mutated genes (Table S2). Eleven of 45 (24%) were mutated in ≥ four samples (Fig. 1B). Mutations in eight genes (*SRSF2*,* ASXL1*,* KMT2D*,* GATA2*,* U2AF1*,* SF3B1*,* BCOR*, and *STAG2*) were enriched in our samples (P value < 0.05 based on a chi square test) compared with mutation frequencies from the Beat AML study [7]. These include multiple genes that are associated with worse prognosis (*SRSF2*,* U2AF1*,* KMT2D*,* ASXL1*,* RUNX1*,* BCOR*,* TET2*).

**Table 1:** Jumping translocation (JT) cohorts in this study

JT Type	Data Source (age, sex)	Disease	Total	Pre-JT* Karyotype “*N*” for normal and “A” for abnormal karyotype	Jumping Translocation (JT) Karyotype											
					Recipient chromosomes involved in JT*** In the number of patients								Extra chromosomal abnormalities besides JT***			
					**Acrocentric Chromosome (p arms)**					**Telomeric Region**	**Centromeric Region**	**Other Region**	0	1	2	≥ 3
					**13p**	**14p**	**15p**	**21p**	**22p**							
1q-JT	JH cohort, median age 76 (46–88 yrs), 11 M / 10 F	AML	14	7 N / 3 A	6	5	9	5	6	2 (1p, 7q, 18p, 18q)	2 (16q)	0	6	5	2	1
		MDS	4	1 N	1	1	1	0	1	3 (2q, 7q, 18q)	2 (16q)	0	1	3	0	0
		MDS -> AML	2	2 N	2	0	0	0	1	0	1 (Yq)	0	1	1	0	0
		MPN	1	NA	1	1	0	0	0	0	0	0	1	0	0	0
	MD Anderson cohort, median age 66 (43–90 yrs), 14 M / 4 F	AML	3	2 N / 1 A	1	2	2	1	0	1 (7p, 9q)	2 (Yq, 7p)	1	2	1	0	0
		CMML -> AML	3	3 N	1	3	3	1	0	2 (4q, Yq)	3 (Yq, 9p/q, 16p, 18q)	0	1	0	2	0
		MDS	6	2 N / 1 A	4	3	1	3	1	3 (4q, 5q, 18q)	4 (Yq, 5p, 7p, 12p, 16p/q, 19p)	2	3	0	1	2
		MDS / T-MDS -> AML	4	1 N / 2 A	3	3	2	1	1	2 (6q, 7q)	1 (Yp)	2	3	0	1	0
		MPN	1	1 N	0	0	1	0	0	0	1 (7p, 9p)	0	1	0	0	0
		MPN -> blast phase	1	1 A	1	0	1	0	1	1 (8p, 17p)	1 (20p)	0	0	0	1	0
	Total number of patients		39	19 N / 8 A	20	19	20	12	11	14	17	5	19	10	7	3
Non 1q-JT**	MD Anderson cohort, median age 43 (40–46 yrs), 3 M / 4 F	AML	4	4 N	0	0	0	1	0	3 (1q, 6p/q, 9q, 10q, 12q, 13q, 14q, 16p)	1 (16p, 17q, 18p)	3	4	0	0	0
		AML -> AML w/ MRC	2	2 A	0	0	0	0	0	2 (5q, 6p, 11p, 17q)	0	1	2	0	0	0
		CMML -> AML	1	1 N	0	0	0	0	0	1 (9q, 20q)	0	1	0	1	0	0
	Total number of patients		7	5 N / 2 A	0	0	0	1	0	6	1	5	6	1	0	0

*The majority of pre-JT specimens had normal karyotypes (approximately 71%). **Non-1q JT cases involved bands of 1p22, 1q25, 3q21, 7p15, 12p13, 15q21, and 21q22, all of which involved telomeric regions as recipient chromosomes. Receipt chromosomes in non-1q JT were different from these in 1q-JT. The majority of receipt chromosomes in 1q-JT involved in the short arms of acrocentric chromosomes (approximately 70%). ***Acrocentric Chromosomes, “Telomeric Regions”, “Centromeric Regions”, “Other Regions”, and “Extra chromosomal abnormalities besides JT” refer to the number of patients. A = Abnormal karyotype; N = Normal karyotype; NA = Data not available; yrs = years. AML: acute myeloid leukemia; AML w/MRC: acute myeloid leukemia with myelodysplasia related changes; CMML: chronic myelomonocytic leukemia; F: female; JT: jumping translocation; M: male; MDS: myelodysplastic syndrome; MPN: myeloproliferative neoplasm; T-MDS: treatment-related myelodysplastic syndrome.

Among the most frequently mutated genes, the most frequently significantly co-mutated gene was *BCOR* (Fig. 1C, Pearson correlation tests). *BCOR* was significantly co-mutated with *KMT2D* and *STAG2*, q-value = 0.01 (Fig. 1C). Two other pairs of genes were significantly co-mutated, one pair is *U2AF1* and *GATA2* and the other pair is *RUNX1* and *SRSF2* (Fig. 1C). *SRSF2* and *RUNX1* have been shown previously to co-mutate in AML [8].

Across the thirteen patients with specimens tested both before and after the JT event, the median variant allele frequencies (VAFs) of mutations in these eleven genes did not change significantly post-JT (P value 0.21 -1.0 based on Wilcoxon rank sum test).

For validation of our mutational findings within the Johns Hopkins (JH) cohort, we collected an additional 25 patients with myeloid malignancies from MD Anderson (MDA) including 18 1q-JT patients and 7 non-1q JT patients (Table 1, S1). Of the 45 mutated genes from the JH-cohort, 17 were observed to be mutated in ≥ one patient in the MDA-cohort. Of the eleven genes most mutated in the JH-cohort, eight were included in the MDA-panel and five were mutated in ≥ two patients in the 1q-JT MDA-cohort (Figure S1). In descending order of frequency, these genes included *RUNX1*,* SRSF2*,* TET2*,* SF3B1*, and *ASXL1*.

Even with a large number of simple/non-complex karyotypes (93.5%) in this study (Table 1/S1), the median overall survival after JT occurrence was 10 months (Fig. 1D, S2). Longer survivals were observed in patients with allogeneic hematopoietic cell transplantation (alloHCT).

Pre 1q-JT patients had a long interval [median = 914-day, range = 105–7539 days] to the first specimens with 1q-JT occurrence from diagnosis (Table S1). The majority of patients had treatments involving hypomethylating agents (HMAs) before JT (Figure S2). For example, case #2 had maintenance azacitidine for > 9-year after alloHCT before he developed 1q-JT. He and other two patients (case #17, 36) also developed an “intermediate” JT-like chromosomal rearrangement, with 1q donor to only one recipient chromosome. Some mutations, such as *BCOR* and *TET2*, have been reported to be associated with being sensitive and having a better response to HMAs [8, 9]. HMAs might be associated with hypomethylating the large pericentromeric heterochromatin region of chromosome 1 [10, 11].

SNP microarray, optical genome mapping, and the NGS data revealed 1q-gain with various proximal breakpoints and the same terminal breakpoint and either one or both homologous chromosomes 1 as JT donor chromosomes (Table S3, Figures S3-5). Except two terminal deletions of two recipient chromosomes (Telomeric 1pter and 18pter regions involving in 1q-JT in case #16), no additional deletions among recipient chromosomes were observed by SNP microarray or optical genome mapping. Combination 1q-gain and the pre-JT gene mutations may cooperate to promote cancer progression and develop more aggressive disease.

This study is the first multi-center cohort having mutation profiles of before and after the 1q-JT events in myeloid malignancies. This study revealed frequent mutations, most of which are associated with worse prognosis. While pre 1q-JT had a long > 2.5-year median interval to the specimens with 1q-JT occurrence, the median overall survival after 1q-JT was 10 months, supporting combination 1q-gain and the pre-JT gene mutations may cooperate to develop more aggressive disease. To our knowledge, our cohort represents the largest number of 1q-JT in myeloid malignancies analyzed to date. Overall, 1q-JT could serve as a poor prognosis biomarker in myeloid malignancies.

### Electronic supplementary material

Below is the link to the electronic supplementary material.

Additional file 1. Supplementary data includes supplementary material and methods, supplementary tables S1-2 and supplementary figure S1-S4

## Data Availability

No datasets were generated or analysed during the current study.
