# Supplementary material for: 1q jumping translocation as a biomarker in myeloid malignancy: frequently mutated genes associated with bad prognosis and low survival
Source: Exp Hematol Oncol. 2024 Aug 1;13:73. doi: 10.1186/s40164-024-00541-3 (PMC11295323; doi:10.1186/s40164-024-00541-3)
Supplement: Supplementary file 1 — Additional file 1. Supplementary data includes supplementary material and methods, supplementary tables S1-2 and supplementary figure S1-S4 [file 40164_2024_541_MOESM1_ESM.pdf]

Supplemental Tables S1-S3

Supplemental Table S1: Jumping translocations (JT) in patients with myeloid malignancies

Sample ID	Pt. ID	Disease	Age/ Sex	JT event	Time to the first 1q JT event (days)	Recipient chromosomes involved in JT			Extra chromosomal abnormalities besides JT
				+ JT, - JT		P-arms of acrocentric chromosomes	Telomeric regions / other genomic regions	Centromeric (peri-) regions	
**Classic 1q-JT cases (case # 1-39)**									
1	1*	MDS	76/M	+	1		2qter; 18qter	16q11.1	der(18)t(9;18)(q13;q23)
				- (After transplant)	256, 640, 2253				
				+	2376			16q11.1	
2	2*	MDS -> AML	66/M	-	-3402, -3037, -2672, …-847				
3				-	-273	14p			
4				+	1	13p; 14p; 21p			
5				+	406	13p; 14p			
6-9				+	636, 749, 962, 1344	13p; 14p; 21p		Yq11.1	
10	3	MDS	88/M	+	1	13p	7qter		
11-12	4	AML	77/F	-	-1013, -714				
13-14				+	1, 126	14p; 15p; 21p			
15	5	MDS	79/F	-	-105				
16		AML		+	1	13p; 22p			r(7)
17	6	MDS	79/M	-	-600				
18				+	1	15p; 21p		16q11.1	der(16;21)(q10;q10)
19				+	230	15p; 21p			
20	7	AML	81/M	+	1	13p; 21p	7qter		
21				+	814	13p; 21p	7qter		+r
22	8	AML	75/M	-	-1702				
23				-	-1586				+8
24				-	-1450				-Y
25				+	1	15p		16q11.1	add(14)(p11.2), der(14;15)(q10;q10)
26-29	9	AML	64/F	-	-954, -922, -828, -587				
30				+	1	15p; 21p; 22p			t(12;19)(q24.1;q13.3) del(12)(q22q24.1)
31				+	215	15p; 21p			t(12;19)(q24.1;q13.3) del(12)(q22q24.1)
32-33	10	AML	80/F	-	-315, -234				
34, 35				+	1, 31	13p; 14p, 22p			
36	11	AML	86/F	-	-1333				
37-39				+	1, 111, 826	14p; 15p; 22p			+8
40				+	584	14p; 15p			+8
41	12	AML	73/M	+	1	13p; 21p			-Y
42, 43	13	AML	62/M	-	-275, -166				+3,del(9)(q13q22)
44				+	1	14p; 15p			+3,+del(8)(q21), del(9)(q13q22),+mar
45	14	AML	67/F	-	-1248				
46				+	1	13p; 14p; 22p			inv(1)(p13q21)
47	15*	MPN	46/F	+	1	13p; 14p			
48				+	230	14p			
49	16	AML	85/M	+	1		1pter; 18pter		
50				+	417		1pter; 18qter		add(18)(q22)
51	17	AML	73/F	-	-22	22p			del(11)(q13q23)
52				+	1	15p; 22p			
53-54	18	AML	79/F	-	-1203, -1133				
55				+	1	13p; 15p			
56	19	MDS	75/F	+	1	14p; 22p	18qter		+8
57-58	19			+	1650, 1776	14p; 21p; 22p	18qter		add(18)(q12)
59-61	20	AML	69/M	+	1, 300, 372	13p, 15p		16q11	
62	21	AML	63/M	-	-448				t(4;8)(q12;q24), +8
63				+	1	15p, 21p, 22p			

64	22	MDS->AML	68/M	+	1	13p, 14p, 15p			+8, +11, +13 from non-JT clones
65	23	MDS	81/F	-	-1505				del(6)(q13q23)
66-72		AML		+	1, 29, 113, 155, 184, 634, 686	13p; 14p; 15p; 22p			del(6)(q13q23) from non-JT clones
73	24	CMML	65/M	-	-490				
74-79		AML		+	1, 34, 70, 98, 169, 188	13p; 14p; 15p; 21p	4qter	9q12; 16p10; 18q10; Yq12	
80	25	AML	90/M	-	-874				
81-82				+	1, 15	13p; 14p; 15p; 21p	/ 2p23	Yq12	del(1)(q32q42)
83	26	AML	70/M	-	-1435				+mar
84-85				+	1, 87	14p		7p10	dup(1)(q12q44) from non-JT clones
86	27	MPN -> blast phase	67/F	-	-7539				+8,del(20)(q11.2)
87-88				+	1, 330	13p; 15p; 22p	8pter; 17pter	20p11	+8,del(20)(q13.1)
89	28	CMML	82/M	-	-1203				
90		AML		+	1	14p; 15p		9p10	der(Y)t(Y;9)(q11.23;q13), add(14)(p11.2)
91	29	MDS	64/M	+	1, 64, 202, 264	14p		7p10; 19p10	+9,+21,del(1)(p13)
92	30	CMML	65/M	-	-349				
93-94		AML		+	1, 123	14p; 15p	Yqter	9q12; 16p10	del(6)(q15q21), der(Y)t(Y;9)(q12;q12)
95	31	MDS	65/M	-	-545				+8,+19
96-98				+	1, 390, 484	13p; 14p; 21p			+8,+19
99	32	MDS	60/M	+	1	13p; 15p; 21p	4qter		
100	33	MPN	45/M	-	-1985				
101-102				+	1, 606	15p		7p10; 9p10	
103	34	MDS	75/M	-	-700				
104-105				+	1, 94	13p	18qter	Yq11; 12p11; 16q11	
106	35	AML	69/M	-	-204				
107-108				+	1, 49	15p	7pter; 9qter		
109	36	T-MDS	64/F	-	-832	21p			t(10;21)(q25;q11.2)
110		T-MDS		+	1	13p; 21p	/ 3p25		t(3;10;21)(q27;q25;q22)
111		AML		+	64	13p; 14p; 21p	7qter / 3p25; 6q26, 18q22		t(3;10;21)(q27;q25;q22),
112		AML		+	108	13p; 14p; 21p	/ 3p25		t(3;10;21)(q27;q25;q22)
113		AML		+	143	13p; 14p; 21p	3p25		t(3;10;21)(q27;q25;q22) del(7)(p12)
114	37	MDS	66/M	-	-1592				
115				+	1		5qter / 4q31.3; 18q21	16p10	
116	38	MDS	43/M	-	-385				
117		AML		+	1		6pter / 7q22; 10q22, 12q15	Yp11	
118-119	39	MDS	57/F	+	1, 180	13p, 14p, 21p, 22p	/ 11q23, 18q21.1	5p11	del(6)(q13),-9,+mar, del(7)(p21),-13,-22

Non 1q-JT cases (case #40-46)

120	40	AML	62/F	-	-554				+del(1)(p13),-3,-18,+3-4mar
121		AML w/ MRC		+ (1q25)	1		5qter, 6pter, 17qter		
122	41	AML	63/F	-	-42				der(3)inv(3)(p25q13.2)inv(3)(q21q26), del(5)(q13q33),+8,+13
123		AML w/ MRC		+ (1p22)	1		11pter / 3q21; 5q13		del(5)(q13q33) from non-JT clones
124	42	CMML	80/F	-	-644				
125		AML		+ (3q21)	1	21p	9qter; 20qter / Xp22.1		add(3)(q13.2)
126	43	AML	32/M	-	-152				
127-129				+ (7p15)	1, 39, 80		1qter; 9qter; 12qter; 13qter. 16pter / 15q26.1		t(X;7)(p10;q10),t(8;17)(p21;q21) from non-JT clones
130	44	AML	33/M	-	-337				
131				+ (12p13)	1		/ 1p13; 7p15; 10q22; 12q13		
132	45	AML	72/M	-	-253				
133-142				+ (15q21)	1, 36, 94, 157, 192, 194, 228, 241, 261, 291		14qter, 6pter, 6qter, / 3p25, 15q24		
143	46	AML	46/F	-	-256				
144				+ (21q22)	1		10qter	16p11; 17q11; 18p11	

Case #1-39 were classic 1q-JT cases and case #40-46 were non 1q-JT cases. AML: acute myeloid leukemia; AML w/MRC: acute myeloid leukemia with myelodysplasia related changes; CMML: chronic myelomonocytic leukemia; F: female; JT: jumping translocation; M: male;

MDS: myelodysplastic syndrome; MPN: myeloproliferative neoplasm; pt.: patient; T-MDS: treatment-related myelodysplastic syndrome; ter: terminal end of chromosome

Table S2: mutations/variants in 45 genes among 1q-JT patients

Genes (n = 45)	Mutations / Variants (number of patients)
KMT2D.chr12	p.P998T, p.R2188C, p.P2210L, p.S4073L, p.Q3475_H3476insQ
IDH2.chr15	p.R140Q (3)
SRSF2.chr17	p.P95L (3), p.P95R (2), p.P95H
RUNX1.chr21	p.R166*, p.R169T, p.D198N, p.R204*, p.R320*, p.E422A
STAG2.chrX	p.A350fs, c.463-1G>C, p.L591fs, p.R1012*, c.1018-1G>C, p.S1058*
BCOR.chrX	p.R1341W, p.I730fs, p.R1234G, p.L1157fs
ASXL1.chr20	p.E797fs, p.G646fs (3), p.L815Q, p.A627fs, p.P699fs, p.E635fs (2), p.R693*
NRAS.chr1	p.G12D, p.G12C, p.G13R
IDH1.chr2	p.R132H, p.R132C, p.R119P
GATA2.chr3	p.M388_E391delinsI, p.S261fs, p.M388_K389del
SGK1.chr6	p.P295L
RECQL4.chr8 **	c.2297-1C>G (2), p.G556D, p.K141T
NUP98.chr11	p.S1067A, p.E948D
NLRP1.chr17	p.R308Q
TP53.chr17	p.Y163C, p.R282W, p.H233P
EP300.chr22	p.L2393V, p.R580Q, p.P748R
NSD1.chr5	p.V2618I, p.G1231E
NOTCH1.chr9	p.H316P, p.A1343V
NOTCH2.chr1	p.V2075M (2)
CREBBP.chr16	p.G1305S
SF3B1.chr2	p.K700E (2)
TET2.chr4	p.C296*, p.N439fs, p.Q831*, p.Q916*, p.K959*, p.E1057fs, p.L1111fs, P1115fs, N1118fs, p.H1219fs, p.V1232fs, R1359G, p.R1440fs,p.W1847*, p.H1881L
DNMT3A.chr2	p.G413fs, p.Y584*
CHEK2.chr22	c.444+1G>A
NF1.chr17	p.S361T, p.I679fs
JAK2.chr9	p.V617F (2)
CBL.chr11	p.C404Y
PTPN11.chr12	p.A72S
ETV6.chr12	p.K421fs, p.N85fs, p.K421*
U2AF1;U2AF1L5.chr21	p.S34F, p.R156H (2), p.Q157P
CARD11.chr7	p.S881G
KRAS.chr12	p.G12D, p.G12S, p.A146P
CEBPA.chr19	p.K298E
KIT.chr4	p.N410Y
RAD50.chr5	p.V315L
GNAS.chr20	p.T415_G423del, p.T415A, p.A436V
EZH2.chr7	p.L671V
MPL.chr1	p.Y591H
BORCS8-MEF2B;MEF2B.chr19	p.P301L
PHF6.chrX	p.R76fs, p.I314T, p.G248D
ZRSR2.chrX	p.C326G
ERBB2.chr17	p.R896H
SETBP1.chr18	p.P1091T
PLCG2.chr16	p.R1224H
DDX41.chr5	p.R525H

**variants in the RECQL4 gene were of unknown clinical significance and germline.

Table S3: SNP microarray and optical genome mapping data for cases with 1q jumping translocations

Case ID	Chr.	Region	SNP microarray data				OGM*
			Copy Number Abnormality	Start	Stop	Size (bp)	
2	1	1q21.1 to q44 (terminal)	Gain	**144,906,508**	249,218,992	104,312,484	Yes
3	1	1q21.2 to q44 (terminal)	Gain	**145,444,556**	249,218,992	103,774,436	
	4	4q24	Loss (including *TET2* gene)	105,986,597	106,390,734	404,137	
	19	19q12 to qter (terminal)	CN-LOH	29,901,465	59,097,160	29,195,695	
7	1	1q21.1 to q44 (terminal)	Gain	**144,853,079**	249,218,992	104,365,913	
	22	22q11.1 to q13.33 (terminal)	CN-LOH	16,114,244	51,511,392	35,397,148	
8	1	1q21.1 to q44 (terminal)	Gain**	**144,861,940**	249,218,992	104,357,052	
	16	16q11.2 to q (whole arm)	CN-LOH	46,450,037	90,274,695	43,824,658	
11	1	1q21.1 to q44 (terminal)	Gain	**144,938,320**	249,218,992	104,280,672	Yes
	8	8pter to qter	Gain	Whole chromosome		N/A	Yes
16	1	1p36.33 to p36.22 (terminal)	Loss	689,189	9,842,576	9,153,387	
	1	1q21.1 to q44	Gain (3-4 Copies)	**145,394,955**	249,218,992	103,824,037	
	18	18p11.32 (terminal)	Loss	13,034	2,228,201	2,215,167	
	21	21q11.2 to q22.3 (terminal)	CN-LOH	14,613,203	48,100,155	33,486,952	

* Optical Genome mapping revealed all copy number variants detected by SNP microarray. No additional structural variants involving chromosome 1 were observed. **Gain without allelic imbalance in the BAF plot. Chr.: chromosome. All locations were based on the hg19 genome builder.

Supplemental Figures S1-S5

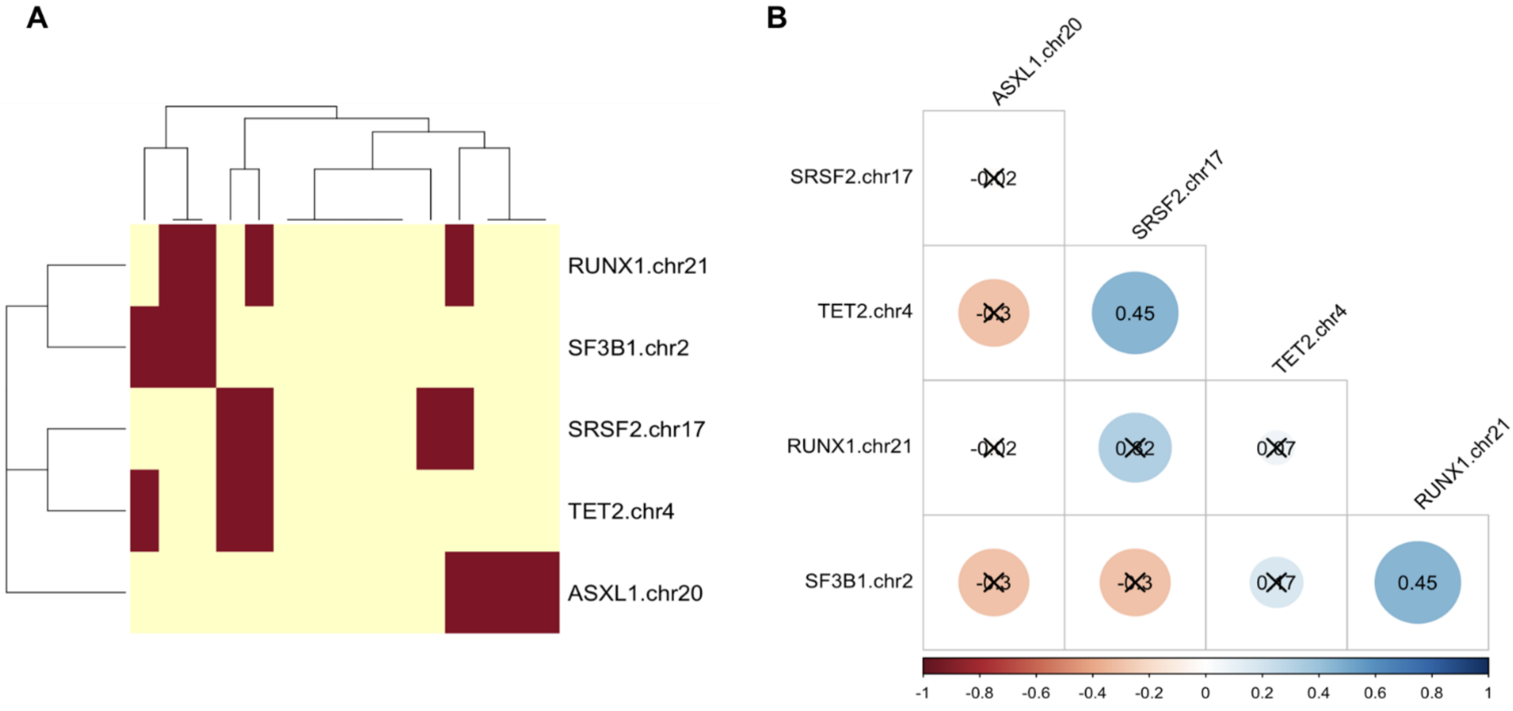

Fig. S1 Mutation profiles of jumping translocations in the MD Anderson cohort. A. Heat map of the common mutated genes in this cohort. B. Correlations in the common mutated genes in this cohort. Numbers in circles indicate Pearson correlation coefficients.

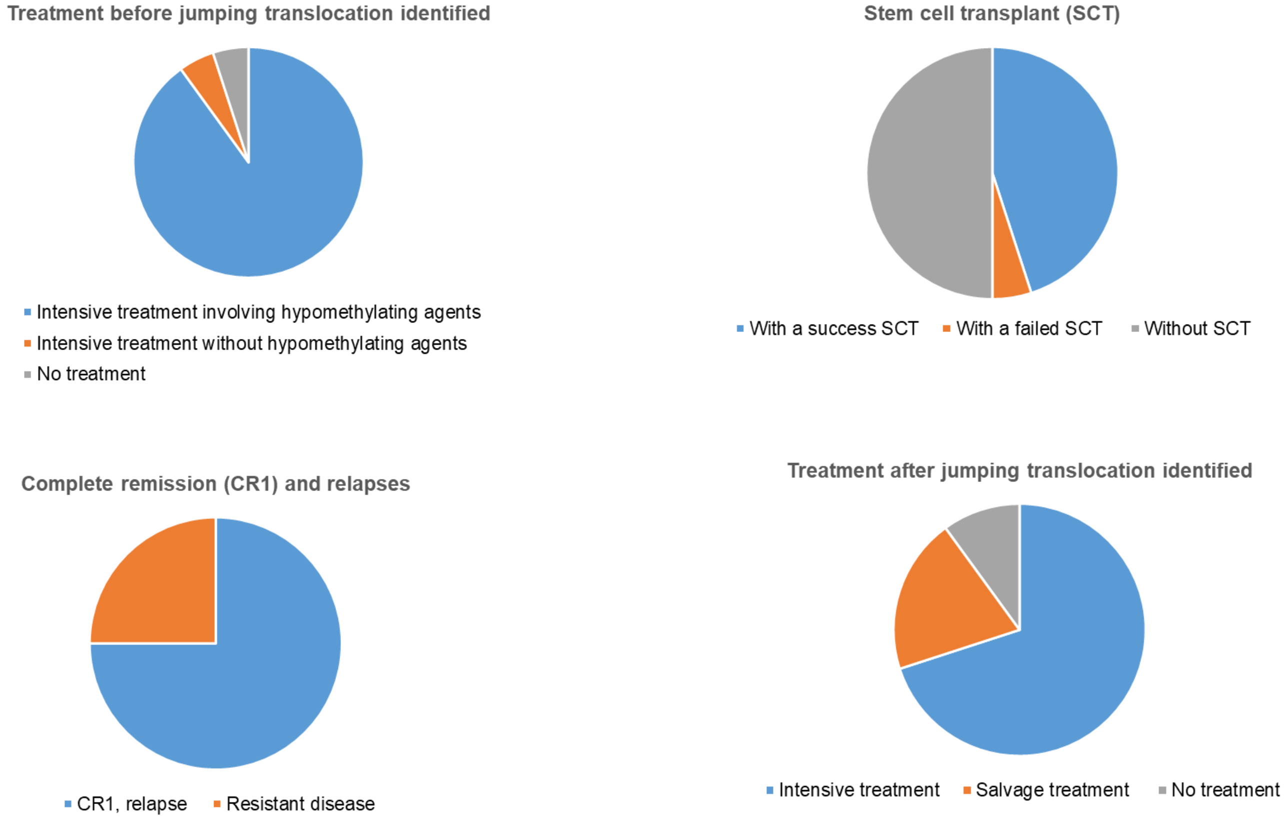

Fig. S2 Treatment information of 1q-jumping translocation patients in this study.

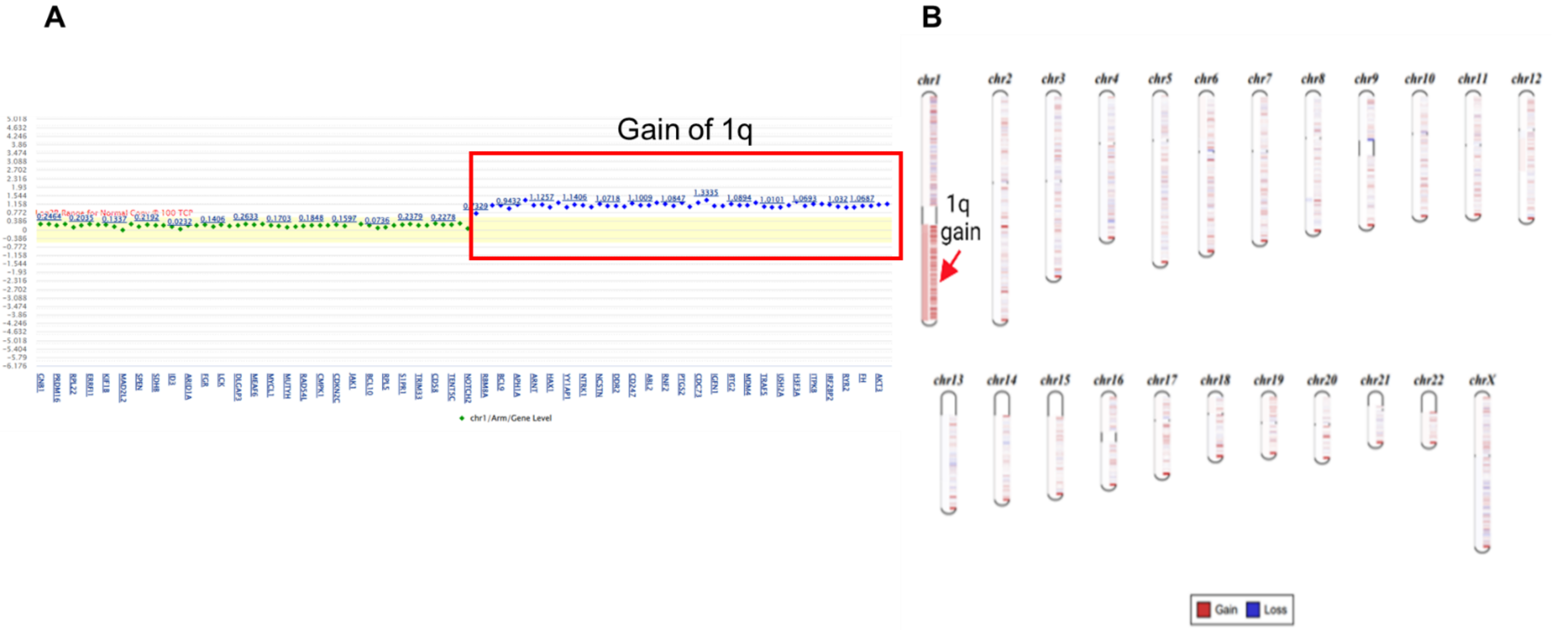

Fig. S3 A. Diagram of copy number variants across entire chromosome 1 from the targeted next-generation sequencing assay to show gain of 1q (indicated by the red box). B. Diagram from CNVkit software version 0.9.6 shows gain of 1q. Red arrow points to the 1q gain.

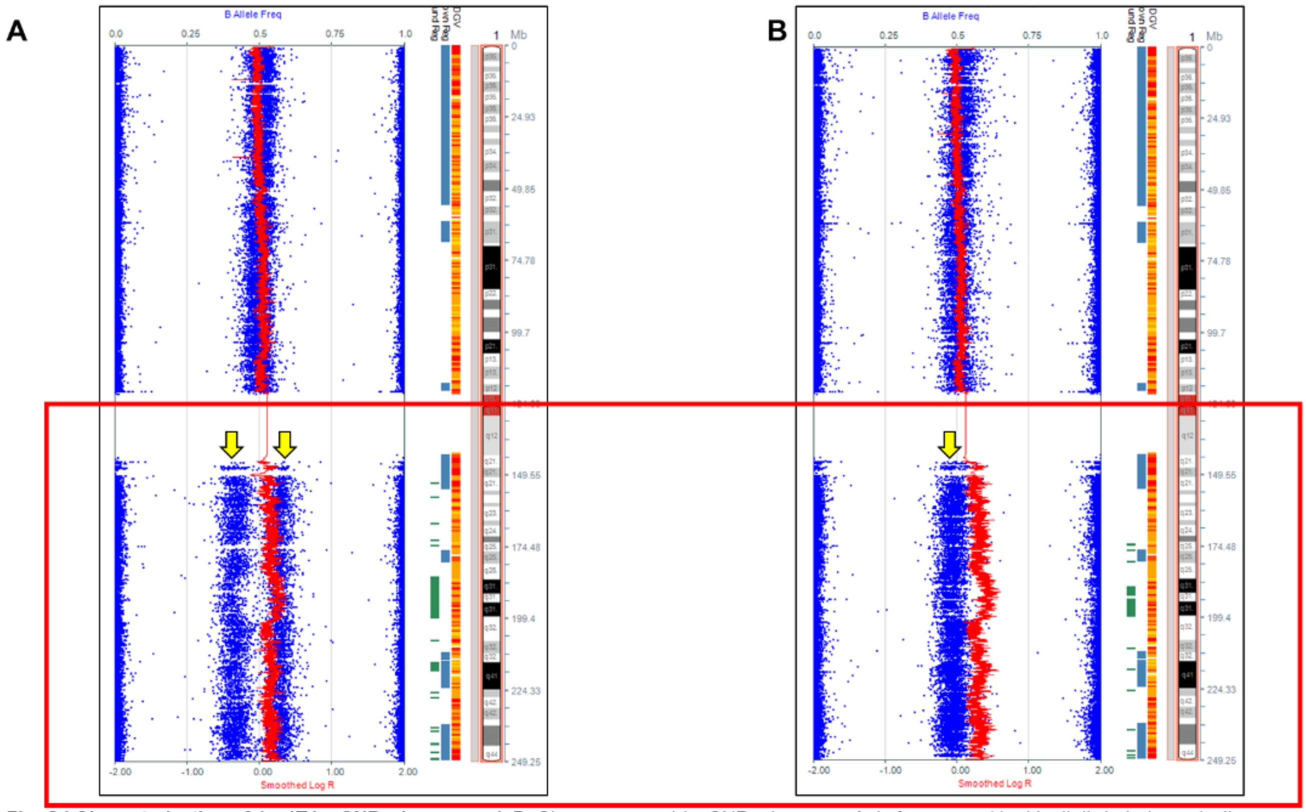

Fig. S4 Characterization of 1q-JT by SNP microarray. A-B. Chromosomes 1 by SNP microarray. A. is from case 11 with allelic imbalance (yellow arrows), suggesting one of chromosomes 1 as the donor chromosome involved in 1q-JT formation. B. is from case 8 without allelic imbalance (yellow arrow) suggesting both homologues chromosomes 1 as donor chromosomes involved in 1q-JT formation.

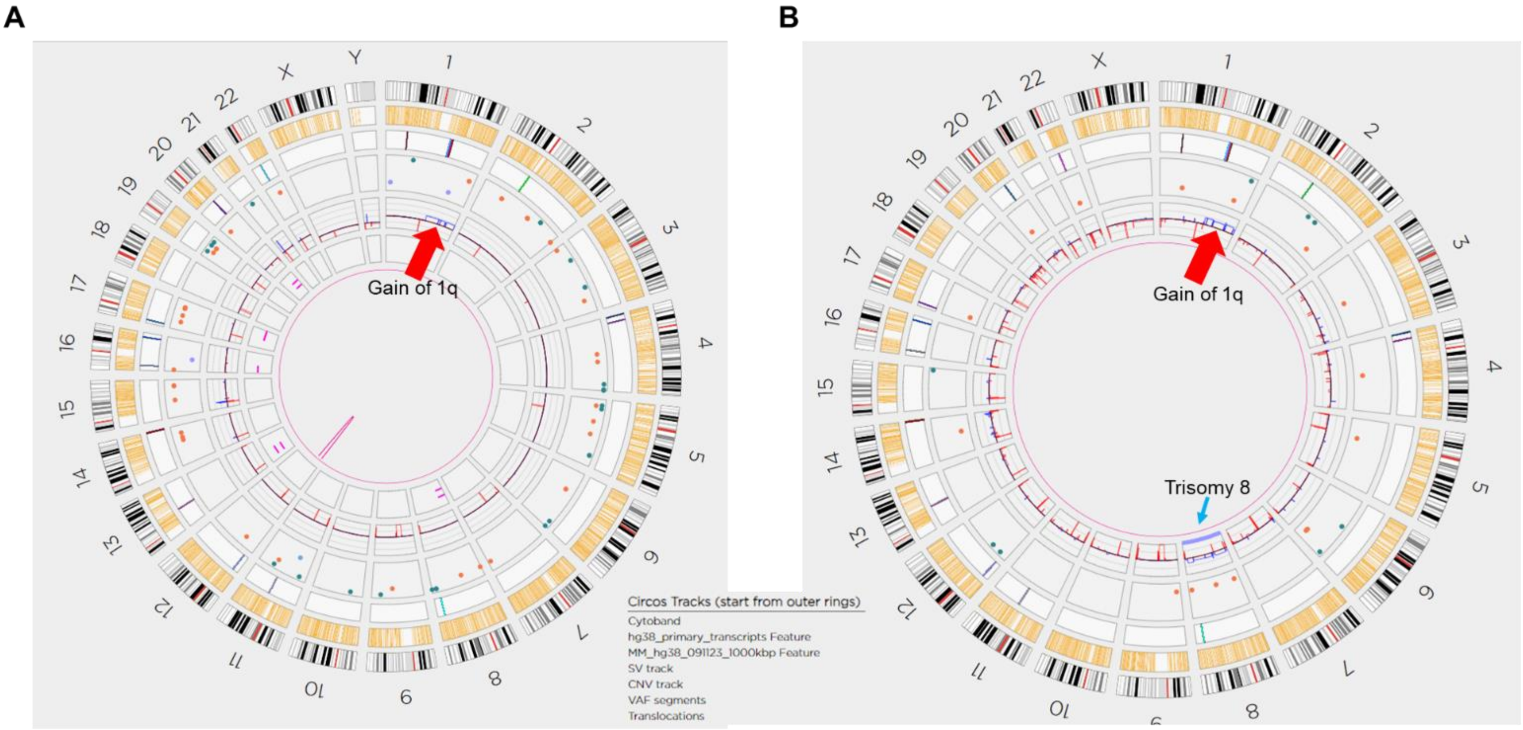

Fig. S5 Characterization of 1q-JT by optical genome mapping. A-B. Chromosomes 1 by optical genome mapping. A is from case 2 showed gain of 1q and no additional structural variants (SVs) involving chromosome 1. B is from case 11 showed gain of 1q, trisomy 8, and no additional SVs involving chromosome 1.

The supplementary Methods

Patients and Samples

This study includes 144 specimens from 46 patients with myeloid malignancies referred to The Johns Hopkins Hospital and The University of Texas MD Anderson Cancer Center from January 1, 2016, to December 31, 2023. These patients had routine diagnostic procedures, including morphologic evaluation, flow cytometry, fluorescence *in situ* hybridization (FISH), conventional chromosome analysis, and/or a targeted next-

generation sequencing (NGS) assay. Disease classification by standard hematopathology practice and delineated by the World Health Organization was based on clinical, morphologic, immunophenotypic, cytogenetic, and molecular genetic features.

Conventional Chromosome Analysis

Conventional G-banded chromosome studies were performed using standard techniques. A minimum of 20 metaphase cells were analyzed from fresh bone marrow aspirate. The abnormal karyotypes were described using the International System for Human Cytogenetic Nomenclature (2020).

Targeted Next-generation sequencing (NGS) mutation assay

NGS was performed in CLIA/CAP-certified molecular diagnostics labs on fresh bone marrow aspirate. For patients 1 through 21, DNA concentration was assessed using the Qubit fluorometer (Thermo Fisher Scientific, Waltham, MA). Library preparation was performed using Kapa Roche (Wilmington, MA) reagents, hybrid capture was performed using Integrated DNA Technologies probes (Coralville, IA), and products were sequenced using NovaSeq (paired-end technology; Illumina, San Diego, CA). The targeted NGS assay used 40,670 Integrated DNA Technologies probes. For a list of covered cancer genes in the targeted NGS assay, see https://pathology.jhu.edu/jhml-services/assets/test-directory/Myeloma-Panel_GeneList_v1.0.pdf. Analysis was performed using human reference sequence genome assembly hg19 (NCBI build

GRCh37/hg19). An in-house variant caller software (MDL VC 10) was used to generate gene variants/mutations from the targeted NGS data. For patients 22 to 46, NGS gene panel includes *ARID1A, ASXL1, ATM, B2M, BAZ2A, BCL10, BCL2, BCL6, BCL7A, BCOR, BIRC3, BLNK, BRAF, BRCC3, BTG1, BTG2, BTK, CARD11, CCND1, CCND3, CCR4, CCR7, CD274, CD28, CD58, CD79A, CD79B, CDKN2A, CDKN2B, CHD2, CHEK2, CIITA, CNOT3, CREBBP, CXCR4, DDX3X, DIS3, DNMT3A, DUSP2, EGR1, EGR2, ELF4, EP300, EWSR1, EZH2, FAM50A, FAS, FAT1, FBXW7, FGFR3, FOXO1, FYN, GNA13, GNAS, GPR183, H1-2, H1-4, H3C2, HRAS, HUWE1, HVNC1, ID3, IDH1, IDH2, IFNGR1, IGLL5, IKZF3, IL2RG, IRAK1, IRF4, IRF8, ITPKB, JAK1, JAK2, JAK3, KIT, KLF2, KLHL6, KMT2D, KRAS, LTB, LYN, MAP2K1, MAP3K14, MAPK1, MAX, MED12, MEF2B, MFHAS1, MYC, MYD88, NF1, NFKB2, NFKBIA, NFKBIE, NOTCH1, NOTCH2, NPM1, NRAS, NSD2, NXF1, P2RY8, PAX5, PIK3CA, PIK3R1, PIM1, PLCG1, PLCG2, PLEKHG5, POLE, POT1, PRDM1, PTEN, PTPN1, PTPN11, PTPRD, RASSF1, RB1, RBMX, RFTN1, RHOA, RIPK1, RPS15, RRAGC, RRAS, S1PR1, S1PR2, SAMHD1, SETD2, SF3B1, SGK1, SMARCA4, SMO, SOCS1, SOX11, SP140, SPEN, SRSF2, STAT3, STAT5B, STAT6, STK11, SYK, TBL1XR1, TCF3, TENT5C, TET2, TMEM30A, TNFAIP3, TNFRSF14, TP53, TRAF2, TRAF3, TRAF6, U2AF1, UBR5, VAV1, XPO1, ZFAT, ZMYM3,* and *ZRSR2*. NGS had coverage (>250x) and mutant allele frequency (>5%).

Whole-Genome SNP Microarray

Single-nucleotide polymorphism (SNP) microarray was performed with DNA extracted from fresh bone marrow specimens by conventional methods (Qiacube). DNA

concentration was assessed using the Qubit fluorometer. The high-resolution microarray platform utilized was the Illumina Infinium CytoSNP-850K version 1.2 BeadChip, containing >850,000 markers (mean spacing, 3.5 kb). BeadChips were processed per manufacturer's guidelines and imaged with the Illumina iScan system. Data were analyzed with the CNV Partition 2.4.4.0 algorithm in GenomeStudio version 2010.3 (Illumina) and KaryoStudio version 1.4.3.0 (Illumina). B-allele frequency and logR signal intensities were used to examine and to identify potentially pathogenic regions of genomic imbalance. All analysis was performed using human reference genome assembly hg19 (GRCh37).

Optical genome mapping (OGM)

OGM was performed on fresh biopsy/aspirates. G3.3 chips were utilized, and samples were processed on the Bionano Saphyr instrument (San Diego, CA, USA). OGM analysis was performed using the Rare Variant Analysis (RVA) and De Novo (DN) pipelines, utilizing the Bionano Access software v1.7.2. CNVs and SVs were manually determined independently by two genetic analysts. All analysis was performed using human reference genome assembly hg19 (GRCh37).
